# Treatment and monitoring of SAPHO syndrome: a systematic review

**DOI:** 10.1136/rmdopen-2023-003688

**Published:** 2023-12-26

**Authors:** Sophie W S Li, Eve Roberts, Christian Hedrich

**Affiliations:** 1 Department of Women's & Children's Health, Institute of Life Course and Medical Sciences, University of Liverpool, Liverpool, UK; 2 Department of Paediatric Rheumatology, Alder Hey Children's NHS Foundation Trust Hospital, Liverpool, UK

**Keywords:** Inflammation, Treatment, Synovitis

## Abstract

**Background and objectives:**

Synovitis acne pustulosis hyperostosis osteitis (SAPHO) is a rare heterogeneous disease of unknown aetiopathology. Externally validated and internationally agreed diagnostic criteria or outcomes and, as a result, prospective randomised controlled trials in SAPHO are absent. Consequently, there is no agreed treatment standard. This study aimed to systematically collate and discuss treatment options in SAPHO.

**Methods:**

Following ‘Preferred Reporting Items for Systematic Reviews and Meta-Analyses’ guidance, a systematic literature search was conducted using PubMed, Scopus and Web of Science databases. Prospective clinical studies and retrospective case collections discussing management and outcomes in SAPHO involving five or more participants were included. Articles not published in English, studies not reporting defined outcomes, and studies solely relying on patient-reported outcomes were excluded.

**Results:**

A total of 28 studies (20 observational, 8 open-label clinical studies) reporting 796 patients of predominantly European ethnicity were included. Reported therapies varied greatly, with many centres using multiple treatments in parallel. Most patients (37.1%) received non-steroidal anti-inflammatory drugs alone or in combination. Bisphosphonates (22.1%), conventional (21.7%) and biological (11.3%) disease-modifying antirheumatic drugs were the next most frequently reported treatments. Reported outcomes varied and delivered mixed results, which complicates comparisons. Bisphosphonates demonstrated the most consistent improvement of osteoarticular symptoms and were associated with transient influenza-like symptoms. Paradoxical skin reactions were reported in patients treated with TNF inhibitors, but no serious adverse events were recorded. Most treatments had limited or mixed effects on cutaneous involvement. A recent study investigating the Janus kinase inhibitor tofacitinib delivered promising results in relation to skin and nail involvement.

**Conclusions:**

No single currently available treatment option sufficiently addresses all SAPHO-associated symptoms. Variable, sometimes descriptive outcomes and the use of treatment combinations complicate conclusions and treatment recommendations. Randomised clinical trials are necessary to generate reliable evidence.

**What is already known on this topic**

- Synovitis acne pustulosis hyperostosis osteitis (SAPHO) is a syndrome encompassing musculoskeletal and dermatological manifestations, for which there are currently no agreed standard treatment protocols.

**What this study adds**

- Observational and open-label clinical studies of treatments for SAPHO report upon a range of drugs including NSAIDs, bisphosphonates and disease-modifying antirheumatic drugs. However, outcome measures vary and symptom control differs between patients, with a combination of therapies often being needed.

**This study might affect research, practice or policy**

- Randomised controlled trials are needed to determine optimum treatment protocols that will address all manifestations of SAPHO. This will require the generation of internationally-agreed classification criteria and outcomes measures.

## Introduction

The clinical syndrome including synovitis, acne, (palmoplantar) pustulosis and hyperostosis osteitis, also known as synovitis acne pustulosis hyperostosis osteitis (SAPHO), constitutes a rare systemic inflammatory disease of currently unknown aetiopathology.^1–4^ SAPHO is characterised by osteoarticular and dermatological features following a waxing and waning course with episodes of remission and exacerbation.^1 2 5^ SAPHO frequently affects the anterior chest wall and axial skeleton, but other regions may be affected.^1 3^ The clinical presentation is heterogeneous, with variable impact on quality of life due to pain, functional impairment and cosmetic alterations.^3 4 6–8^ While SAPHO typically affects adults, it can also be present in children and young people. Notably, because of overlapping clinical features, especially sterile osteitis, SAPHO may be closely related to chronic non-bacterial osteomyelitis (CNO), its severe form chronic recurrent multifocal osteomyelitis (CRMO),^2 3 5^ and pustulotic arthro-osteitis (PAO)^9^ which may all represent ‘limited forms’ of SAPHO.

In the absence of reliable epidemiological data, a prevalence of <1/10 000 has been estimated.^10 11^ Reports from all geographic regions are available in the literature, and a female predilection (approximately 2.2:1) has been suggested (by at least 23 here included reports from European, Asian, African and Australian populations).^3 4 6 7 12–30^

In the current absence of widely accepted and independently validated diagnostic and classification criteria, diagnosing SAPHO relies on the presence of clinical features (including sterile bone inflammation, arthritis, inflammatory skin disease) and the exclusion of differential diagnoses (such as infections, tumours, Langerhans cell histiocytosis).^2^ Several sets of diagnostic or classification criteria have been proposed, of which the ones developed by, Kahn *et al* (1994 modified in 2003) and Benhamou *et al* are most commonly used across the literature (table 1).^9 31–35^ Notably, classification criteria (other than diagnostic criteria) are developed to define clinically homogeneous disease cohorts for the inclusion in clinical trials. They are aiming for high specificity at the expense of sensitivity.^36^ Alongside radiographic imaging (plain radiographs, CT), bone scintigraphy, MRI, whole-body MRI and positron emission tomography (PET) CT are considered key diagnostic tools.^31^ Notably, scintigraphy is limited by high exposure to radiation, reduced ability to discriminate between growth plates and bone lesions (in young patients), and the inability to interpret extraosseous tissues.^37^ Plain radiographs and CT scans provide an understanding of structural changes and damage.^38^ MRI and PET CT enable early detection of bone lesions prior to structural damage, and provide information on soft tissue involvement.^31^

**Table 1:** Proposed criteria for SAPHO

Criteria	Inclusion	Exclusion	Quality
Kahn and Khan^9^	Any of the three presentations are sufficient for diagnosis: Chronic recurrent multifocal osteomyelitis Usually sterile Spine may be involved With or without skin condition Acute, subacute, or chronic arthritis associated with any of the following: Palmoplantar pustulosis Pustular psoriasis Severe acne Any sterile (or with presence of *Propionibacterium acnes*) osteitis (one localisation is sufficient, including spondylodiscitis) associated with any of the following: Palmoplantar pustulosis Pustular psoriasis Psoriasis vulgaris Severe acne		Diagnostic
Kahn^34 35^ (modified 2003)	Bone/joint involvement associated with palmoplantar pustulosis and psoriasis vulgaris Bone/joint involvement associated with severe acne Isolated sterile (except *P. acnes*) hyperostosis/osteitis (adults) Chronic recurrent multifocal osteomyelitis (children) Bone/joint involvement associated with chronic bowel diseases	Infectious osteitis Tumours of bone Non-inflammatory condensing lesions of bone	Classification
Benhamou *et al*^32 33^	One of the following four is sufficient to diagnose, in absence of an exclusion feature: Osteoarticular manifestations of severe acne Osteoarticular manifestations of palmoplantar pustulosis Hyperostosis with or without dermatosis Chronic recurrent multifocal osteomyelitis involving axial or peripheral skeleton, with or without dermatosis Sometimes reported: Possible association with psoriasis vulgaris Possible association with inflammatory enterocolopathy Features of ankylosing spondylitis Presence of low virulence bacterial infections	Septic osteomyelitis Infectious chest wall arthritis Infectious palmoplantar pustulosis Palmoplantar keratodermia Diffuse idiopathic skeletal hyperostosis Osteoarticular manifestations of retinoid therapy	Diagnostic

SAPHO, synovitis acne pustulosis hyperostosis osteitis.

The exact molecular pathophysiology of SAPHO remains unknown. Most studies addressing disease mechanisms focus on paediatric CNO/CRMO.^39–41^ Because of clinical overlaps, some authors suggested that SAPHO may be a ‘late stage’ or the adult form of CNO/CRMO, and the result of ongoing innate immune activation that, over time, may result in the activation of adaptive immune mechanisms and pathological activation of effector T lymphocytes. Adaptive immune activation may, through the release of effector T cell-derived cytokines (such as IL-12, IL-17A, IL-23), result in the development of skin and nail changes frequently seen in SAPHO.^42 43^ Because of overlapping features, such as skin and sacroiliac involvement, some authors also suggested that SAPHO may be closely linked to spondyloarthropathies, another effector T-cell-mediated disease. However, other than in spondyloarthropathies, a strong association with HLA-B27 has not been reported in SAPHO.^25 30^ Similar to CNO/CRMO in the paediatric population, associated autoimmune diseases among the SAPHO patients have been reported.^5 8 44^

**Figure 1:**
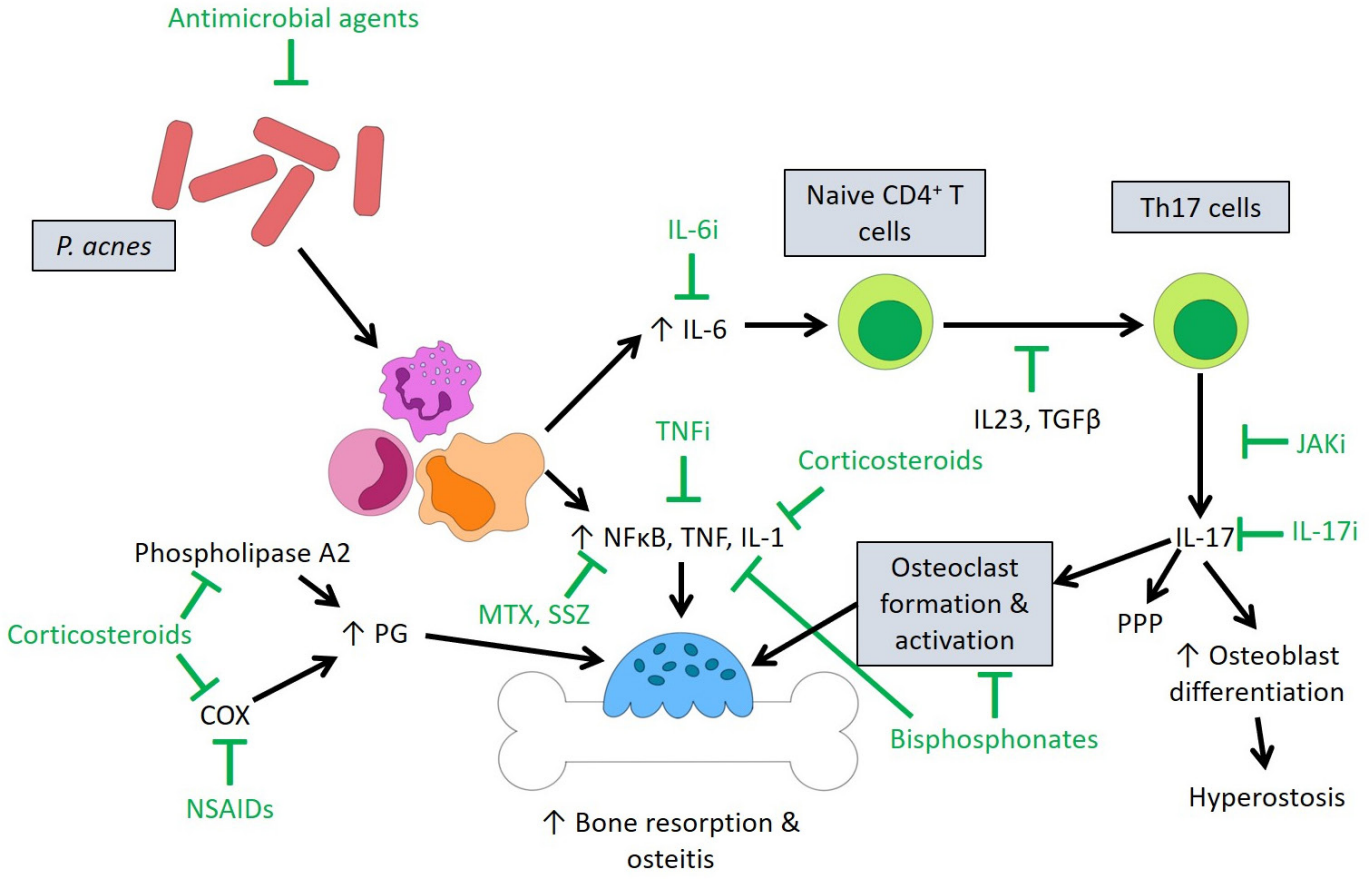
The molecular pathophysiology and treatment options for SAPHO (adapted from Goenka *et al*).^43^ The exact aetiology of SAPHO is uncertain but likely links immune dysfunction with genetic predisposition and infectious triggers. Dysregulated activation of transcriptional regulatory factors (NFkB) and proinflammatory cytokines (IL-1, IL-6, IL-17, IL-23, TNF) results in dysregulated inflammatory responses and sterile osteitis. Proinflammatory cytokines IL-1, IL-6, TNF and IL-17, and prostaglandins (PG) promote osteoclast formation, resulting in increased bone remodelling. Furthermore, IL-17 may also be implicated in palmoplantar pustulosis (PPP) and psoriasis. Treatments reported in the literature target various molecular and cellular contributors to SAPHO. Antimicrobial agents target skin colonisation with Propionibacterium acnes. Biological TNF inhibitors (TNFi), IL-6 receptor blockers (IL-6i) and IL-17 inhibitors (IL-17i) limit inflammatory cytokine signalling, while JAK inhibitors (JAKi) may inhibit proinflammatory cytokine expression and the differentiation of effector Th17 T-cells. Bisphosphonates cause osteoclast apoptosis, thereby inhibiting bone resorption, but also have anti-inflammatory effects through the correction of imbalances proinflammatory and anti-inflammatory cytokine expression. Corticosteroids inhibit phospholipase A2 and COX enzymes, reducing PGs. Additionally, corticosteroids alter expression of NFκΒ-dependent proinflammatory cytokines. NSAIDs inhibit COX enzymes reducing PG production. Methotrexate (MTX) and sulfasalazine (SSZ) reduce proinflammatory cytokine expression.^39 43 56^ NSAIDs, non-steroidal anti-inflammatory drugs; SAPHO, synovitis acne pustulosis hyperostosis osteitis.

Considering clinical characteristics and suspected molecular and cellular mechanisms involved in CNO/CRMO and SAPHO, several treatments have been suggested to control pain and inflammation. Treatments reported in the literature include antimicrobial agents, non-steroidal anti-inflammatory drugs (NSAIDs), corticosteroids, bisphosphonates, conventional disease-modifying antirheumatic drug (cDMARDs) and biological (bDMARDs)^6 41^ (figure 1). However, because of its rarity and the limited understanding of its molecular pathophysiology, evidence is limited to small open-label studies and case series.

This manuscript systematically reviews the available literature, including case series and clinical studies investigating treatment response in SAPHO patients (including five or more patients).

## Methods

This review was conducted in accordance with ‘Preferred Reporting Items for Systematic Reviews and Meta-Analyses’ guidelines for systematic reviews.^45^

### Eligibility criteria

Clinical studies (case series, prospective and retrospective cohort studies, clinical trials) discussing treatment outcomes, including adverse effects, in at least five patients were included.

Articles were excluded for at least one of the following reasons:

- Articles not published in English.
- Studies without discussion of treatment.
- Outcomes not reported and/or absent discussion of side effects/adverse events.
- Solely patient-reported outcomes in questionnaires outside of the clinical setting and physician questionnaires.
- Less than five patients involved including case reports.
- Reviews, including systematic reviews.
- Incomplete presentations of SAPHO, that is, adult CNO/CRMO without skin involvement or PAO.

### Information sources

An electronic search conducted on the databases PubMed, Scopus and Web of Science between 11 February 2023 and 12 February 2023. Keywords were chosen considering drug classes and treatments noted in existing literature for SAPHO (online supplemental table S1). Reports on ‘incomplete’ forms of SAPHO, such as PAO and adult CNO/CRMO, were excluded because it remains unclear whether they are, indeed, part of the same disease entity.^9 46–51^

10.1136/rmdopen-2023-003688.supp1 Supplementary data

### Study selection

The study selection was performed by SWSL supported by CH. Records were identified and filtered to include English language publications only. Search results were exported to EndNote V.X9, duplicates were removed using the EndNote V.X9 duplicate finder tool. Records were then screened by title and abstract. Main reasons for exclusion were previously undetected duplicates, manuscript that did not report treatment outcomes, publications with fewer than five patients, and inappropriate manuscript types (including systematic reviews, meta-analyses and letters). Next, full text manuscripts were retrieved. Eligibility was assessed for each paper following the inclusion and exclusion criteria detailed. Only those meeting eligibility criteria were included for analysis.

### Data collection

Relevant data were extracted including study design and observation period, aims, patient inclusion and exclusion criteria, number of participants, demographics, treatment(s), methods and reported outcomes, and adverse effects. Outcome measures were diverse and consequently presented as reported in each study. Paper strengths and limitations were considered alongside study type and methods to assign an evidence level.

## Results

### Literature search

The existing literature mostly comprised observational studies reporting a diverse set of treatments considering variable (mostly descriptive) outcomes.^6 52^ Evidence from randomised controlled trials (RCTs) was limited to a Chinese study, which had to be excluded from this review due to publication language (Chinese).^53^

**Figure 2:**
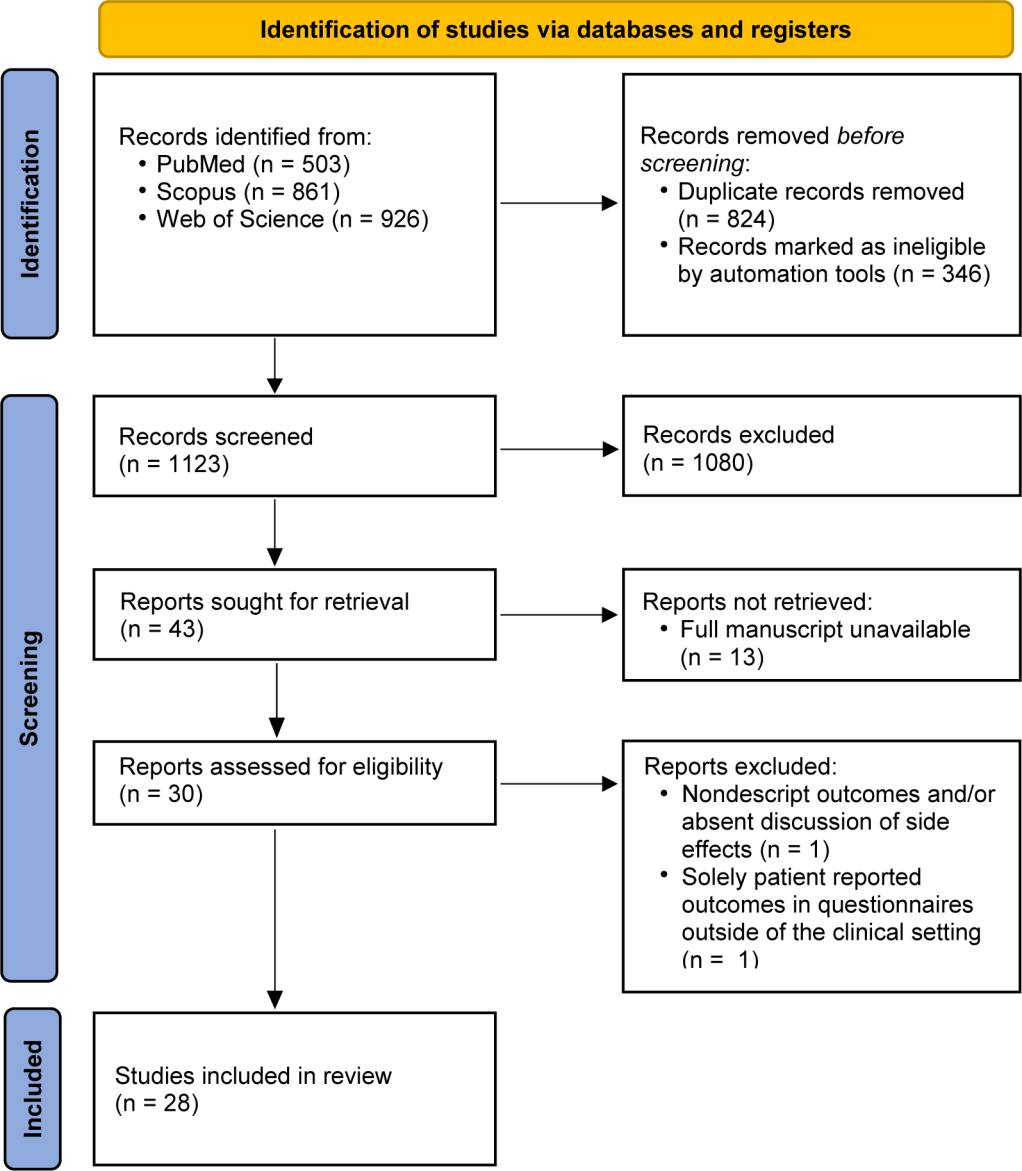
PRISMA diagram displaying search results, exclusion and inclusion of manuscripts.^45^ PRISMA, Preferred Reporting Items for Systematic Reviews and Meta-Analyses.

An initial search delivered 2290 records, of which 1123 manuscripts remained after removing duplicates and non-English publications that underwent title and abstract screening. Of these, 1080 manuscripts were excluded applying exclusion criteria. Remaining manuscripts (N=43) were accessed for a full-text screen. Thirteen publications were removed as they were conference abstracts without an associated full text manuscript (remaining N=30), and two additional publications were removed based on exclusion criteria. Finally, 28 studies were included in full-text review (figure 2).

### Demographic information and reported outcomes

The 28 studies selected for full review included 796 patients, aged 5–84 years. Twenty-five papers detailed sex distribution (including n=553/796, 69.5% of patients) with approximately 2.2:1 female predominance (female n=374/553, 67.6%; male n=179/553, 32.4%) (online supplemental table S2).

Sixteen manuscripts originated from Europe, eight from China and two from Australia. Remaining studies were conducted in Tunisia (N=1) and Israel (N=1). Nine papers detailed ethnicity (including n=444/796, 55.8% of patients). Of these, the majority were White Caucasians (n=223/444, 50.2%), followed by Asian ethnicity (n=202/444, 45.5%, of whom 188/202, 93.1% were Chinese). A minority of reported patients were of black ethnicity (n=19/444, 4.3%).

Outcome measures applied across 28 studies were heterogeneous and varied in quality and quantity (online supplemental table S3,S4). Most studies focused on outcomes relating to osteoarticular manifestations, such as pain improvement via Visual Analogue Scale (VAS) pain scores (10/28 studies, 35.7%), ‘other’ patient-reported outcomes (8/28, 28.6%) and need for treatment escalation (4/28, 14.3%). Global disease activity was most commonly assessed through the Bath Ankylosing Spondylitis Disease Activity Index (BASDAI, 7/28, 25%), Ankylosing Spondylitis Disease Activity Score (ASDAS, 2/28, 7.14%) and number of flares after treatment initiation (3/28, 10.7%). Other measures included patient questionnaires for osteitis-associated complaints, Physician Global Assessment, Health Assessment Score (HAS), and physician assessed osteitis and skin disease activity. Evidence of osteoarticular involvement on MRI was the most commonly reported imaging outcome (6/28, 21.4%), followed by changes on plain X-rays (3/28, 10.7%), and CT including cone beam CT (CBCT, an imaging modality developed specifically for imaging teeth, jaws and temporomandibular joint) (3/28, 10.7%).^23^ Laboratory inflammatory markers reported usually included Erythrocyte Sedimentation Rate (ESR) (10/28, 35.7%) and C reactive protein (CRP) (9/28, 32.1%). Fewer studies addressed skin and nail manifestations (10/28, 35.7%). Most of these studies reported descriptive measures (6/10, 60%), however, Nail Psoriasis Severity Index (NAPSI, 2/10, 20%) and Palmoplantar Pustulosis Area and Severity Index (PPPASI, 3/10, 30%) were also used.

### Treatments

Several treatments were reported across the 28 studies either alone or, most frequently, in combination.^4–6 23 26^ Likely because of variable involvement of various organ systems (predominantly skin and bone), symptomatic improvement was frequently associated with using multiple drug therapies in parallel. Bisphosphonate use was reported in 17/28 (60.7%) studies (pamidronate 12/17; alendronate and zoledronic acid 1/17; not specified 4/17).^2 3 5–7 12 13 15 16 19–21 23 26 29 30 54^ NSAIDs (11/28, 39.3%), cDMARDs (10/28, 35.7%), including methotrexate, sulfasalazine and azathioprine, and bDMARDs, including TNF inhibitors (TNFi, 11/28, 39.3%) and IL-17 blockers (IL-17i, 2/28, 7.1%%), were discussed. One study reported the use of Janus kinase (JAK) inhibition with tofacitinib.^18^ No serious adverse events were reported for treatments used (online supplemental table S5).

### Antimicrobial agents

Historically, infections (especially with *Propionibacterium acnes*) had been considered as a pathomechanistic feature of SAPHO.^8 52^ As a result, 85 patients from 6 studies were treated with antimicrobial agents none of which resulted in sustained remission^4 12 19 25 26 28^ (figure 1). Azithromycin and doxycycline were used in several studies despite negative bacteriological bone cultures, likely to address skin colonisation associated with acne. Unsurprisingly, only a minority of patients (11/55, 20%) reported (time-limited) symptomatic improvement.^4 12 19 26 28^ In patients with positive bacterial cultures for *P. acnes* in bone biopsies, antimicrobial treatment improved osteitis lesions and reduced skin disease activity after 16 weeks. However, disease activity returned to baseline following discontinuation of antimicrobial treatment.^25^ In one study, despite negative or unknown bacterial cultures for *P. acnes*, antimicrobial therapy with clindamycin in combination with NSAIDs (below) resulted in clinical improvement with persistent pain resolution (4/4, 100%) and improved imaging findings (osteosclerosis n=2; osteitis n=2; osteolysis n=1) after 3 months or 4 months treatment. Another patient had resolution of sternoclavicular joint swelling and tenderness after 8 months of treatment.^4^ However, it remained unclear whether effects were due to antimicrobial agents and/or NSAIDs.

Only few and mild adverse effects were reported, including not further specified ‘intolerance’ (doxycycline n=2; azithromycin n=1).^12 25^

### Non-steroidal anti-inflammatory drugs

NSAIDs are frequently used in rheumatology to modulate pain, inflammation, and osteoclast activity through inhibition of prostaglandin synthesis^41 55 56^ (figure 1). Across studies, NSAIDs were usually used as first-line treatment to provide pain relief in SAPHO.^6 8 52^ A small proportion of SAPHO patients achieved partial or full remission in response to NSAID treatment alone, including 20% (4/20) of patients in a small Belgian cohort.^2 5 12 19 26 29 30^ The majority of studies, however, reported inadequate effects or incomplete improvement.^5 21 30^ Of those studies, reporting efficacy of NSAIDs, most focused on pain reduction.^2 4 6 8 28^ One study also reported improved radiological and dermatological features (in addition to pain resolution) in patients treated with a combination of the antimicrobial agent clindamycin and lornoxicam.^4^

Side effects of NSAIDs included gastrointestinal symptoms, but no severe toxicity.^21 26^

### Corticosteroids

Corticosteroids inhibit phospholipase A, which results in reduced prostaglandin E production affecting pain, inflammation and osteoclast function. Furthermore, they alter the expression of NFκB-dependent proinflammatory cytokines and enhance anti-inflammatory cytokine expression (including IL-10)^41 56^ (figure 1). Long-term use of corticosteroids is limited by side effects that, among others, include loss of bone density, thinning of skin, weight gain and the development of iatrogenic Cushing’s syndrome, infections, etc.^57^ Corticosteroids can be applied systemically or locally (topical use on the skin, joint injections).^52^

Across 7 studies, a total of 56 SAPHO patients were treated with corticosteroids. Topical corticosteroids were used for dermatological manifestations, with psoriasis vulgaris resolution in one case when taken alongside adalimumab and eosin.^5^ Intra-articular corticosteroid injections were reported by four studies. Three studies suggested intra-articular corticosteroids to be effective, providing greater osteoarticular improvement when compared with oral therapy.^12 17 26^ One study failed to identify positive effects on osteitis (p=0.06) using 20 mg intra-articular triamcinolone but suggested benefit for arthritis management.^17^

Systemic corticosteroids were used with transient and/or partial improvement in 26 individuals from three studies.^2 26 29^ Favourable outcomes were reported in individuals when corticosteroids were given in combination with either adalimumab (psoriasis vulgaris resolution, 1/1), *Tripterygium wilfordii Hook F* (a traditional Chinese herb with immunosuppressive effects: muscle oedema regression, 4/4; subcutaneous soft tissue swelling regression, 1/1; bone marrow oedema (BMO) regression, 2/4; periosteitis regression, 1/2; temporomandibular joint synovial thickening regression, 3/3) or methotrexate (cortical/medullary lysis on CBCT, 1/1).^5 23^ In a small Chinese cohort, corticosteroid monotherapy led to regression of muscle and BMO (both 2/3, 66.7%), subcutaneous soft tissue swelling (1/1) and temporomandibular arthritis (1/1).^23^

While no adverse events or severe side effects were reported in these included studies, long-term follow-up is lacking, and concerns remain in relation to drug safety and recognised corticosteroid-associated complications.^58^

### Bisphosphonates

Bisphosphonates inhibit osteoclast-mediated bone resorption and have anti-inflammatory effects, reconstituting the balance between proinflammatory and anti-inflammatory cytokines.^3 6 7 13 15 52^ A total of 176 patients were treated with bisphosphonates across 17 studies. Notably, reported effects of bisphosphonates are mostly limited to bone inflammation with no consistent improvement of cutaneous lesions across the literature.^59 60^

Treatment with bisphosphonates improved osteoarticular disease in a majority of patients across studies.^2 3 6 7 12 13 15 16 19–21 23 26 29 30^ This included patients refractory to NSAIDs and corticosteroid injections.^12 13 26^ Active agents, administration methods and doses vary between studies (table 2). Four manuscripts did not disclose specific bisphosphonates used, route of administration, nor dose.^23 26 29 30^ Improvement was described as partial or complete pain resolution.^12 13 26 29 30^ Where available, MRI confirmed rapid and sustained improvement of spinal BMO in two studies including 44 patients (for 37 of whom MRI studies were available).^7 23^ Somewhat unexpectedly, one open-label study also reported beneficial effects of pamidronate on skin lesions, preventing recurrence of pustulosis in 9/10 patients (90%) refractory to oral corticosteroids, colchicine, methotrexate, sulfasalazine or infliximab.^13^ However, NSAID use was permitted as concomitant treatment, which may have had additional effects.^13^ Others did not confirm effects on the skin.^15^

**Table 2:** Bisphosphonate use across studies

Bisphosphonate administered	Dosing information	Study
Undisclosed	Undisclosed	Hayem^11^ Skrabl-Baumgartner^29^ M Wang *et al*^23^ Zwaenepoel and Vlam^23 26 29 30^
Intravenous pamidronate	Undisclosed	Maatallah *et al*^19^ Wu *et al*^2^ Yap *et al*^2 19 54^
	1 mg/kg/day for three consecutive days	C Li *et al*^7^*† Maccora *et al*^5^* Kerrison *et al*‡^3 5 7^
	Single infusion (30 mg)	Van Doornum *et al*^21^
	Single infusion per course (60 mg)	Amital *et al*§^13^
	Infusion of 60 mg/day for three consecutive days	Colina *et al*^15^ Guignard *et al*^16^¶ Solau-Gervais *et al*^15 16 20^
	Single infusion per course (180 mg)	Aljuhani *et al*^12^**
Intravenous zoledronic acid	Undisclosed	Yap *et al*^54^
	5 mg/year	Huang *et al*^6^
Oral alendronate	70 mg/week	Huang *et al*^6^

*No maximum daily dose specified.

†At baseline and at 3 months.

‡Maximum 30 mg daily, repeat every 3 months if necessary.

§Single infusion given within 1 hour, additional infusions given within 1 month if no response and at 4 months if partial response, further infusions thereafter given at least 4 months apart.

¶During disease exacerbations; first patient had single 60 mg infusion per course.

**Number of infusions per course not specified; 12 patients had several courses.

Across studies, pamidronate was the most frequently chosen bisphosphonate (n=122/176 patients, 69.3%; 12/17 studies, 70.6%). Pamidronate was effective in early disease, but may also have a role in late-stage otherwise treatment refractory SAPHO.^15 16^ Intravenous pamidronate effectively controlled osteoarticular lesions, with most patients experiencing partial or complete pain control.^13 15 16 19 20^ Some studies reported reduction of osteolytic lesions coinciding with complete clinical, laboratory and radiological remission.^2 23^ In one study, pain relief was associated with reduced need for other medications, including NSAIDs.^16^ A study in paediatric SAPHO patients suggested persistent effects of pamidronate with improved activity and well-being.^3^

Though not included across all international treatment plans for paediatric CNO/CRMO because of lacking information on anti-inflammatory effects,^61 62^ alendronate and zoledronic acid have been used in SAPHO in a Chinese study and an Australian study.^6 54^ Symptomatic relief has been reported in 16/18 (88.9%) patients in the Chinese study (70 mg alendronate per week (15 recipients) or 5 mg zoledronate annually (3 recipients). However, the benefit from bisphosphonate alone is difficult to ascertain as 17/18 (94.4%) received concomitant therapy with NSAIDs, DMARDs, low-dose corticosteroids or TNFi.^6^ Alendronate monotherapy was effective in one Australian patient.^6^ Three patients from the same study received either pamidronate or zoledronic acid, which did not improve bone involvement.^54^

Bisphosphonate associated side effects were in line with previous reports and included influenza-like symptoms and/or timely limited feverish episodes,^3 5 7 12 16^ headaches and mild hypocalcaemia^7^ in patients receiving pamidronate. Gastrointestinal disturbances were infrequently reported in Chinese cohorts. However, one patient received cDMARDs alongside alendronate.^6 7^ Notably, one study from Italy reported no side effects in a cohort of 14 patients.^15^ Taken together, bisphosphonates appear safe and promising, particularly with spinal BMO.^7 23 54^

### Conventional (non-biologic) DMARDs

cDMARDs are usually used when symptoms persist despite NSAID treatment.^52^ Across 10 studies, 173 patients were treated with variable effects. Methotrexate and sulfasalazine reduce inflammatory cytokine expression, thus potentially correcting the imbalance between proinflammatory and anti-inflammatory signals.^63^ Methotrexate and sulfasalazine were most commonly used.^6 12 19 23 26–30 54^ Two studies reported colchicine use (in 34 patients) with little or no improvement.^12 26^ Azathioprine, leflunomide and cyclosporine were reported in a few individual patients.^6 29 30 54^

Three studies suggested beneficial effects of methotrexate on osteoarticular and cutaneous involvement.^19 26 54^ Other studies suggested that using NSAIDs or corticosteroids alongside methotrexate may be more effective than methotrexate alone.^6 23 29^ Overall, complete inefficacy of methotrexate was relatively uncommon (11/54 patients from 6 studies, 20.4%).^6 12 19 26 29 54^ Among these, 8/13 patients relapsed following good initial response,^54^ and 2 switched to other cDMARDs without additional benefit.^29^

Sulfasalazine was associated with minimal or partial improvement of pain, palmoplantar pustulosis and acne.^12 26^ Some patients refractory to NSAIDs experienced resolution of osteoarticular pain.^19^ One patient had over 17 years sustained benefit as demonstrated by Physician Global Assessment scores.^54^

Overall, cDMARDs were tolerated without severe side effects. Most publications did not report side effects beyond gastric discomfort in patients receiving methotrexate or sulfasalazine.^6^ One study suggested that methotrexate (without concomitant corticosteroids) may be associated with metabolic syndrome.^28^

### Biological DMARDs

bDMARDs are used in otherwise treatment refractory patients. Cytokine blocking strategies are used to (at least partially) correct the cytokine imbalance associated with SAPHO (figure 1). 2 5 6 12 14 19 23 24 27 29 30 54 64

A total of 90 patients from 11 studies were treated with TNFi. Overall, reported effects were positive.^6 14 19 27 54^ In one case where adalimumab lost efficacy with worsening skeletal and cutaneous symptoms after initial pain and palmoplantar pustulosis improvement, add-on cDMARD therapy with methotrexate, sulfasalazine and eventually cyclosporine led to symptom relief.^6^ Reports of inefficacy were few (n=5/90, 5.56%),^6 12 14 29^ and included an individual who had initial clinical response but ultimately had to add on cDMARD treatment^6^ and two patients who initially benefited from infliximab before switching to alternative TNFi for recurrence of bone pain.^14^

Most studies reported partial or complete clinical response, including reduction of pain, disease activity as measured with BASDAI and other osteoarticular presentations including sacroiliitis.^2 5 6 14 30 54 64^ Skin improvement was described in four Chinese individuals.^64^ The use of etanercept was associated with complete clinical and laboratory/imaging remission in most patients of a small Chinese cohort (7/10, 70%), but did not allow discontinuation of concomitant therapy in a French study.^2 27^ Improvement was sustained across several studies, including pain control following treatment discontinuation,^64^ and preventing joint and spinal damage progression (8/11; 73%) of patients continuing treatment for at least 1 year.^54^ Infliximab led to persistent physician-assessed benefit for 3 years in one individual.^12^ Adalimumab remained efficacious with reduced BASDAI for 22 months in another individual.^14^

Side effects were rare in patients treated with TNFi. Nine cases (9/90, 10%) of psoriasiform lesions were noted across two studies, including paradoxical psoriasis, existing palmoplantar pustulosis exacerbations and urticaria.^14 64^ Late onset of skin manifestations during the disease course were associated with increased risk of exacerbated lesions under TNFi in the Chinese study.^64^ Most paradoxical lesions resolved after discontinuing TNFi and introduction of non-biological therapy.^14 64^ However, at least some of these potential adverse events may have been associated with the natural course of disease in some patients as SAPHO is associated with psoriasiform skin disease.

Additional cytokine blocking agents targeting IL-6 (a factor during effector Th17 T-cell differentiation) or the IL-23/IL-17 axis have been considered for SAPHO because of clinical overlaps with psoriasis and spondylarthritis.^23 24 54^ Nine patients were treated across three studies from China, France and Australia. Blockade of the IL-6 receptor with tocilizumab failed to induce bone lysis remission on CT in a Chinese SAPHO patient.^23^ Outcomes in response to IL-17 blockade with secukinumab or ixekizumab were disappointing as none of the three Australian patients reached full and sustained remission.^54^ Ustekinumab targets IL-12/23 and led to cutaneous benefit in 33.3% (1/3) of a small French cohort but was not reported to benefit bone features; secukinumab had benefited 66.7% (2/3) who had not received ustekinumab in the same study.^24^ One case of paradoxical psoriasis (2/9 patients, 22.2%) was reported for each secukinumab and ustekinumab.^24^

### Protein kinase inhibitors

JAKs are involved in cytokine receptor signalling, and the differentiation and activation of effector Th17 cells (figure 1). Because of clinical overlaps between SAPHO, psoriasis and spondyloarthropathies, they may be promising treatment options in patients refractory to other treatments.

One study investigated effects of the JAK inhibitor tofacitinib on dermatological manifestations in 13 Asian female SAPHO patients (12 weeks of 5 mg tofacitinib, twice daily).^18^ Results were positive with significant benefit for nail lesions, palmoplantar pustulosis and associated quality of life. While a reduction of systemic inflammatory parameters was observed (CRP and ESR), no information was provided on bone involvement.

One patient experienced acute tonsillitis during the treatment period. No severe adverse events were reported.

### Other treatments

*Tripterygium wilfordii Hook F* is a Chinese herbal medicine used to treat immunological disorders.^6 22^ The mechanisms underlying its effects are diverse and remain largely unknown. It has been suggested to affect effector T-cell function, monocyte phenotypes, and proinflammatory and anti-inflammatory cytokine expression.^65^ Lacking regulatory approval, its potential toxicity and side effects limit clinical applications in the Western world. Three Chinese studies reported 41 SAPHO patients treated with *Tripterygium wilfordii Hook F*. Treatment associated with improved disease activity (measured by ASDAS and BASDAI) and reduced pain when compared with baseline across two small cohorts.^22 23^ Results were less convincing in the third study, including lack of response in one patient.^6^ Only one of the three studies reported side effects with elevated transaminases (ALT levels) in 14/30 patients (46.7%), of whom one individual had to stop treatment.^22^

In addition to systemic anti-inflammatory treatment, three studies reported patients receiving topical treatments for skin involvement, including psoralen in combination with ultraviolet light radiation (PUVA), retinoids (systemically or topically), betamethasone and calcipotriol (a vitamin D derivative containing ointment).^4 26 30^ Of the two studies reporting patient numbers, four patients were treated, however, treatment efficacy was only discussed in one patient reporting palmoplantar pustulosis improvement following treatment with topical corticosteroids and PUVA.^4^ Lastly, Xiang *et al* suggested a possible association between tonsillectomy and improved cutaneous and osteoarticular manifestations, as ostealgia and palmoplantar pustulosis significantly improved with reduced VAS and PPPASI scores in 6/7 and 6/6 patients, respectively.^66^

## Discussion

The molecular and cellular pathophysiology of SAPHO is incompletely understood, complex and likely multifactorial, involving genetic predisposition and environmental impacts.^52^ Our current, limited understanding suggests a combination of dysregulated innate and adaptive immune mechanisms resulting in clinically variable pictures characterised by skin and bone inflammation.^42^

While some reports suggested the presence of *P. acnes* in bone lesions,^52^ these likely represent skin contaminants.^67^ Indeed, *P. acnes* is a common skin contaminant across biospecimen, especially in adults.^68^ However, *P. acnes* may still affect disease expression and phenotypes as a skin commensal. It may contribute to proinflammatory cytokine expression, including TNF, IL-1, IL-8^7 52^ and the IL-23/Th17 axis.^52^ Based on studies primarily in paediatric CNO/CRMO, bone inflammation in SAPHO is thought to result from disrupted innate immune mechanisms, including NRLP3 inflammasome expression and assembly. Imbalanced cytokine expression may increase osteoclast activity and bone remodelling.^56^ Skin disease, on the other hand, may be the result of adaptive immune dysregulation and the differentiation and activation of effector T cell phenotypes.^39 43^

As a result, several classes of drugs have been applied in SAPHO to control inflammation:

- Antimicrobial agents have been considered because of reports (incorrectly) suggesting *P. acnes* as a causative pathogen^4 12 19 25 26 28^ or a contributing environmental factor.^52^ Unsurprisingly in an autoimmune/inflammatory disease, antimicrobial agents were largely ineffective, especially long-term after their discontinuation. Initial improvement in some patients may have been due to immunomodulating effects.^25^
- NSAIDs have been used because of their analgesic effects. They also have (mild) effects on inflammasome assembly and osteoclast activation.^55 56^ While NSAIDs provide pain relief, they are less effective in extensive bone involvement and for the treatment of skin inflammation.^52^ Thus, they may be applied as first-line treatment in the absence of contraindications and supplemented by add-on therapies if not effective.
- Corticosteroids reduce prostaglandin production by inhibiting phospholipase A2, thereby inhibiting osteoclast activity. Furthermore, corticosteroids reduce the expression of proinflammatory NF-κB-regulated genes while increasing the expression of immunoregulatory cytokines.^56^ While long-term use is prohibited by side effects, they can be considered for induction of remission and/or as bridging therapy, for example, until cDMARDs are developing efficacy.
- Bisphosphonates have been used to treat SAPHO for their effects on osteoclasts and their ability to correct cytokine imbalances.^3 7 13 15 52 56^ Bisphosphonates, especially pamidronate, may additionally suppress production of IL-1β, IL-6 and TNF, reducing chronic inflammation.^3 7 13 15 52^ Bisphosphonates were particularly effective to reduce pain and terminate bone inflammation in SAPHO, while no convincing effects were seen on skin inflammation.^7 12 13 15 23 26 29 30^
- The cDMARDs methotrexate and sulfasalazine can correct proinflammatory and anti-inflammatory cytokine imbalances and have, therefore, successfully been used for the treatment of chronic arthritis and inflammatory skin diseases.^56^ Several reports on the use of methotrexate for bone and skin involvement in SAPHO suggest efficacy,^19 26 54^ while sulfasalazine delivered less promising results.^12 26^
- Several bDMARDs have been used to (partially) correct the cytokine imbalance associated with SAPHO.^43 69^ TNF is a proinflammatory cytokine implicated in autoimmune dysfunction and dysregulated osteoclast function.^64 69^ TNFi correct proinflammatory and immune modulatory cytokine imbalances. They have been successfully used for the treatment of clinically related psoriasis and spondylarthropathies.^2 5 6 14 27 30 54 64^ In SAPHO clinical benefits of TNF inhibitors include resolution of bone inflammation and associated pain as well as skin disease.^56^ In addition to TNF, also IL-17 and IL-23 have been implicated in related autoimmune conditions and may be involved in osteoblast differentiation, pathological bone remodelling and skin involvement in SAPHO.^69^ Somewhat unexpectedly, limited reports on IL-17 (secukinumab, ixekizumab) and IL-12/23 (ustekinumab) blockade documented limited effects on both bone and skin inflammation in SAPHO.^24 54^ Similarly, blockade of IL-6 signalling, a cytokine involved in effector Th17 cell differentiation, had limited effects.^23^

Notably, most aforementioned treatments, individually, had limited effects on all organ systems affected. Especially effects on cutaneous manifestations of SAPHO were somewhat disappointing. Thus, one study examined the JAK inhibitor tofacitinib for the treatment of cutaneous lesions, nail changes and associated quality of life without severe adverse effects.^18^ As dermatological lesions are associated with psychosocial burden, it’s necessary to further explore the role of JAK inhibitors.

Notably, treatment regimens varied not only between studies but also between geographic regions (table 3). While no obvious differences in relation to geographical region (associated predominating ethnicities) and treatment responses were identified, differential effects may still be present. Notably, three Chinese studies investigated effects of *Tripterygium wilfordii Hook F*, an anti-inflammatory herb that acts through several compounds including triptolide.^22^ Lacking medical licensing in Western medicine, unknown mechanisms of action, and an incompletely understood side effect spectrum preclude this medication from routine use.

**Table 3:** Treatments studied by geographical region

Origin of paper	Treatments studied
Australia	Bisphosphonates, NSAIDs
China	Anti-TNFα agents, bisphosphonates, cDMARDs, corticosteroid, interleukin-targeted treatments, NSAIDs, tofacitinib, tonsillectomy, TwFH
Europe	Antibiotics, anti-TNFα agents, bisphosphonates, cDMARDs, corticosteroids, NSAIDs
Israel	Bisphosphonate, NSAIDs
Tunisia	Antibiotics, anti-TNFα agents, bisphosphonate, cDMARDs, NSAIDs

cDMARDs, conventional DMARDs; NSAIDs, non-steroidal anti-inflammatory drugs.

Studies in rare diseases are frequently characterised (and limited) by small sample size, retrospective character, observational or descriptive outcomes, open-label design and other factors limiting their impact.^70–72^ To address these limitations in SAPHO, we defined the evidence levels of studies included in this systematic review using definitions suggested by Ackley *et al* (table 4).^73^ Of the 28 studies included here, 20 were observational in nature, and 8 were open-label clinical trials. As a result, evidence levels assigned were overall relatively low (level 6: n=19; level 4: n=1; level 3: n=8). Robust evidence from RCTs is lacking (online supplemental tables S4 and S6).

**Table 4:** Definitions of evidence levels

Evidence level	Definition	Studies reaching evidence level
1	Evidence from a systematic review or meta-analysis of all relevant RCTs (randomised controlled trial) or evidence-based clinical practice guidelines based on systematic reviews of RCTs or three or more RCTs of good quality that have similar results	0
2	Evidence obtained from at least one well-designed RCT (eg, large multisite RCT)	0
3	Evidence obtained from well-designed controlled trials without randomisation (ie, quasi-experimental)	8
4	Evidence from well-designed case–control or cohort studies with quantitative outcome measures	1
5	Evidence from systematic reviews of descriptive and qualitative studies (meta-synthesis)	0
6	Evidence from a single descriptive or qualitative study; patient reported outcome measures	19
7	Evidence from the opinion of authorities and/or reports of expert committees	0

RCT, randomised controlled trial.

Further key challenges with data interpretation include the absence of internationally agreed case definitions, consistent inclusion/exclusion criteria, defined and validated disease-specific outcome measures, aforementioned small patient numbers, and the frequent use of concomitant treatments that are likely caused by the clinical variability of disease and multiple organ involvements that require various treatments (frequently in combination).

A potential limitation of this review relates to inconsistent nomenclature across the literature, which may have led to the exclusion of studies. Because of the broad clinical spectrum and inconsistent nomenclature (including ‘adult CNO/CRMO’, ‘PAO’, ‘sternocostoclavicular hyperostosis (SCCH)’, etc, which may be limited forms of SAPHO or related disorders), only manuscripts reporting cohorts of ‘complete’ SAPHO patients were considered. Notably, manuscripts reporting CNO/CRMO were not included because it is currently under debate whether CNO and SAPHO are distinct entities.^9 47 48^ Although the authors argue that CNO/CRMO and SAPHO may be considered ‘extremes’ on a spectrum of inflammatory bone diseases, and SAPHO may represent a ‘late stage’ presentation of CNO/CRMO, this systematic review focused on ‘complete’ SAPHO syndrome.^43 47 48 51^ Indeed, in a recent review, Leerling *et al* described adult CNO/CRMO as a subtype within a wider disease spectrum. Authors report that the presence of joint and skin pathology increases the likelihood for physicians to distinguish SAPHO from CNO. Because approximately 50% of physicians managing adult CNO/CRMO distinguish between CNO/CRMO and SAPHO, some SAPHO patients in the literature may have been missed in the systematic review presented here.^47^ Therapeutic approaches to CNO/CRMO are summarised and discussed elsewhere, and similarities exist in the treatment across the CNO/CRMO/SAPHO spectrum usually with NSAIDs considered first-line treatment options, and cDMARDs (eg, methotrexate or sulfasalazine), bisphosphonates and TNFi reserved for severe and/or treatment-refractory cases.^41 47 48 56^ Similarly, opinions vary whether SAPHO and PAO constitute the same disease.^9 46 49 50^ Proposed PAO criteria (Sonozaki *et al*^74^) differ from commonly used SAPHO criteria (summarised in table 1) in that PAO can be limited to PPP and CNO.^46^ This supports the proposal of PAO being a subset within SAPHO, with patients with only PPP and arthralgia sometimes being treated as SAPHO.^49 50^ These clinical differences were a key argument for Yamamoto when suggesting to distinguish PAO and SAPHO.^49^ Notably, the term PAO has primarily been used in reports from Japan,^47^ potentially suggesting contrasting presentations of CNO/CRMO/SAPHO spectrum disorders between Western and East Asian cohorts.^50 51^ Notably, PAO patients (other than CNO/CRMO and SAPHO patients reported in Western cohorts) frequently experience concurrent focal infections, such as tonsillitis and/or arthro-osteitis,^46 50^ which results in surgical approaches to treatment, for example, tonsillectomy.^50 75 76^ Distinct treatments included potassium iodide,^77^ granulocyte and monocyte adsorption apheresis.^78^ Despite this, treatment similarities are seen, with a multicentre study showing many receiving NSAIDs, cDMARDs and antimicrobials.^50^ This is reflected in other papers treating with cyclosporine^50 79^ and adalimumab.^80^ An IL-23 inhibitor, guselkumab, has also been reported.^80–82^ Similar arguments have been made around SCCH being a SAPHO subset and part of the CNO spectrum, and two cases have been reported with success in pain and functional improvement.^83^

Pain scores (VAS) were commonly reported which are complicated by subjective pain experiences of individual patients and a multidimensional experience of pain that may vary between SAPHO patients with comparable disease activity.^84^ Potentially more objective outcome measures include laboratory tests for inflammatory markers. However, systemically increased inflammatory parameters, clinical disease activity and disease burden do not correlate closely in all patients.^14 25 66^ Lastly, differences between ethnic groups (with differential representation between geographical regions) and sexes have also not been considered in studies available to date. Considering ethnic diversities in some Western countries especially, ethnicity across study populations cannot be simply estimated, which limits reliable conclusions in relation to associations between ethnicity and outcomes.

## Conclusions

Several treatment options exist for SAPHO, however none of them are equally effective for all organ systems involved. The absence of internationally agreed and validated diagnostic and classification criteria as well as agreed outcome measures, the small sample size of studies, and variable therapeutic approaches, with many patients requiring multiple treatments, challenge data interpretation and recommendations. Bisphosphonates may achieve rapid remission of bone inflammation and associated pain, cDMARDs and TNFi are effective for bone and (to some extent) skin involvement, while JAKi may be an option especially in otherwise treatment refractory skin and nail involvement. Further studies (especially double blinded prospective RCTs) are urgently needed to improve the evidence base of SAPHO treatment.
