# Supplementary material for: Treatment and monitoring of SAPHO syndrome: a systematic review
Source: RMD Open. 2023 Dec 26;9(4):e003688. doi: 10.1136/rmdopen-2023-003688 (PMC10753757; doi:10.1136/rmdopen-2023-003688)
Supplement: Supplementary data [file rmdopen-2023-003688supp001.pdf]

Table S1: Search strategy

**Database**	**Date last searched**	**Terms searched**	**Number of results**
PubMed	11/02/2023	(sapho) AND (“treatment*” OR “therap*” OR “management*” OR “care” OR “NSAID*” OR “nonsteroidal anti-inflammatory” OR “DMARD*” OR “cDMARD*” OR “MTX” OR “methotrexate” OR “SSZ” OR “bDMARD*” OR “TNF” OR “tumor necrosis factor” OR “antibiotic*” OR “azithromycin” OR “doxycycline” OR “clindamycin” OR “sulfamethoxazole” OR “trimethoprim” OR “corticosteroid” OR “bisphosphonate*” OR “pamidronate” OR “IL-17i” OR “IL-1i” OR “IL-6i” OR “interleukin” OR “inhibitor” OR “JAKi” OR “adalimumab” OR “apremilast” OR “certolizumab” OR “etanercept” OR “ixekizumab” OR “leflunomide” OR “piroxicam” OR “plaquenil” OR “hydroxychloroquine” OR “prednisolone” OR “sulfasalazine” OR “secukinumab” OR “zoledronic acid”)	503
Scopus	11/02/2023	(sapho) AND (“treatment*” OR “therap*” OR “management*” OR “care” OR “NSAID*” OR “nonsteroidal anti-inflammatory” OR “DMARD*” OR “cDMARD*” OR “MTX” OR “methotrexate” OR “SSZ” OR “bDMARD*” OR “TNF” OR “tumor necrosis factor” OR “antibiotic*” OR “azithromycin” OR “doxycycline” OR “clindamycin” OR “sulfamethoxazole” OR “trimethoprim” OR “corticosteroid” OR “bisphosphonate*” OR “pamidronate” OR “IL-17i” OR “IL-1i” OR “IL-6i” OR “interleukin” OR “inhibitor” OR “JAKi” OR “adalimumab” OR “apremilast” OR “certolizumab” OR “etanercept” OR “ixekizumab” OR “leflunomide” OR “piroxicam” OR “plaquenil” OR “hydroxychloroquine” OR “prednisolone” OR “sulfasalazine” OR “secukinumab” OR “zoledronic acid”)	861
Web of Science	12/02/2023	(sapho) AND (“treatment*” OR “therap*” OR “management*” OR “care” OR “NSAID*” OR “nonsteroidal anti-inflammatory” OR “DMARD*” OR “cDMARD*” OR “MTX” OR “methotrexate” OR “SSZ” OR “bDMARD*” OR “TNF” OR “tumor necrosis factor” OR “antibiotic*” OR “azithromycin” OR “doxycycline” OR “clindamycin” OR “sulfamethoxazole” OR “trimethoprim” OR “corticosteroid” OR “bisphosphonate*” OR “pamidronate” OR “IL-17i” OR “IL-1i” OR “IL-6i” OR “interleukin” OR “inhibitor” OR “JAKi” OR “adalimumab” OR “apremilast” OR “certolizumab” OR “etanercept” OR “ixekizumab” OR “leflunomide” OR “piroxicam” OR “plaquenil” OR “hydroxychloroquine” OR “prednisolone” OR “sulfasalazine” OR “secukinumab” OR “zoledronic acid”)	595

Table S2: Study demographics

Study reference (authors)	Female sex (%)	Ethnicity / Study Country (if ethnicity data unavailable)
Aljuhani et al. **[1]**	73.17	• White (n= 40)<br>• Black (n = 1)
Amital et al. **[2]**	70.00	• Sephardic (of Northern African descent) (n= 4)<br>• Ashkenazi (of Eastern European descent) (n= 6)
Assmann et al. **[3]**	53.33	Germany
Abdelghani et al. **[4]**	100.00	France
Colina et al. **[5]**	71.43	Italy
Guignard et al. **[6]**	80.00	France
Hayem et al. **[7]**	58.33	• European White (n= 106)<br>• North African (n= 12)<br>• Black African (n = 1)<br>• French West Indies (n = 1)
Huang et al. **[8]**	62.50	China
Jung et al. **[9]**	73.17	Germany
Kerrison et al. **[10]**	100.00	UK
Li et al. **[11]**	100.00	Asian
Li et al. **[12]**	Undisclosed	Chinese
Li et al. **[13]**	66.67	China
Hurtado-Nedelec et al. **[14]**	82.76	France
Maatallah et al. **[15]**	60.87	Tunisia
Maccora et al. **[16]**	40.00	Caucasian
Matzaroglou et al. **[17]**	60.00	Greece
Przepiera-Będzak et al. **[18]**	86.96	Caucasian
Skrabl-Baumgartner et al. **[19]**	60.00	Austria
Solau-Gervais et al. **[20]**	92.31	France
Van Doornum et al. **[21]**	100.00	Australia
Wang et al. **[22]**	60.00	China
Wang et al. **[23]**	61.54	China
Wendling et al. **[24]**	80.00	France

Wu et al. **[25]**	37.50	Chinese
Xiang et al. **[26]**	Undisclosed	China
Yap et al. **[27]**	Undisclosed	• Caucasian (n=20)<br>• Asian (n=1)
Zwaenepoel et al. **[28]**	76.19	Belgium

Ethnicities stated as presented in original manuscripts due to heterogeneity in terminology.

Table S3: Outcome measures

Outcome measure	Number of studies (%)	References
Symptomatic improvement, osteoarticular		
VAS	10	Li et al. [13]<br>Hayem et al. [7]<br>Colina et al. [5]<br>Guignard et al. [6]<br>Solau-Gervais et al. [20]<br>Li et al. [11]<br>Przepiera-Będzak et al. [18]<br>Ben Abdelghani et al. [4]<br>Wang et al. [22]<br>Xiang et al. [26]
**Need for other treatments (analgesia including NSAIDs; anti-inflammatory therapy)**	4	Kerrison et al. [10]<br>Colina et al. [5]<br>Guignard et al. [6]<br>Ben Abdelghani et al. [4]
**Other patient reported pain improvement (decrease in pain intensity or duration)**	8	Wu et al. [25]<br>Kerrison et al. [10]<br>Matzaroglou et al. [17]<br>Huang et al. [8]<br>Aljuhani et al. [1]<br>Amital et al. [2]<br>Maatallah et al. [15]<br>Van Doornum et al. [21]

**Improvement otherwise undefined**	5	Wu et al. [25]<br>Maccora et al. [16]<br>Zwaenepoel et al. [28]<br>Maatallah et al. [15]<br>Skrabl-Baumgartner et al. [19]
**Symptomatic improvement, cutaneous manifestation**		
NAPSI	2	Li et al. [11]<br>Xiang et al. [26]
**PPPASI**	3	Li et al. [11]<br>Wendling et al. [24]<br>Xiang et al. [26]
**Presence of new paradoxical skin lesions**	1	Li et al. [12]
**Improvement otherwise undefined**	6	Wu et al. [25]<br>Matzaroglou et al. [17]<br>Maccora et al. [16]<br>Huang et al. [8]<br>Maatallah et al. [15]<br>Skrabl-Baumgartner et al. [19]
**Functional improvement**		
BASFI	3	Li et al. [13]<br>Ben Abdelghani et al. [4]<br>Wang et al. [22]
**HAQ**	2	Jung et al. [9]<br>Wang et al. [22]
**BASMI**	1	Li et al. [13]
**ASQoL**	1	Wang et al. [22]
**EQ-5D**	1	Wang et al. [22]
**SF-36**	1	Wang et al. [22]
**DLQI**	1	Li et al. [11]
**Disease activity**		

Exacerbation frequency; interval duration between exacerbations	3	Kerrison et al. [10]<br>Amital et al. [2]<br>Guignard et al. [6]
**BASDAI**	7	Li et al. [13]<br>Li et al. [11]<br>Przepiera-Będzak et al. [18]<br>Jung et al. [9]<br>Ben Abdelghani et al. [4]<br>Wendling et al. [24]<br>Wang et al. [22]
**ASDAS**	2	Li et al. [13]<br>Wang et al. [22]
**Patient questionnaire for osteitis complaints**	1	Jung et al. [9]
**PGA**	2	Yap et al. [27]<br>Wendling et al. [24]
**HAS**	1	Assmann et al. [3]
**Osteitis activity, physician assessed**	1	Assmann et al. [3]
**Skin activity, physician assessed**	1	Assmann et al. [3]
**Blood markers**		
ESR	10	Wu et al. [25]<br>Li et al. [13]<br>Assmann et al. [3]<br>Zwaenepoel et al. [28]<br>Skrabl-Baumgartner et al. [19]<br>Solau-Gervais et al. [20]<br>Li et al. [11]<br>Przepiera-Będzak et al. [11]<br>Jung et al. [9]<br>Wang et al. [22]

**CRP (including hs-CRP)**	9	Wu et al. [25]<br>Li et al. [13]<br>Zwaenepoel et al. [28]<br>Skrabl-Baumgartner et al. [19]<br>Solau-Gervais et al. [20]<br>Li et al. [11]<br>Przepiera-Będzak et al. [18]<br>Jung et al. [9]<br>Wang et al. [22]
**Serum interleukins (IL6, IL8, IL18, IL23)**	2	Wu et al. [25]<br>Przepiera-Będzak et al. [18]
**Total immunoglobulin and autoantibody serum levels**	1	Hurtado-Nedelec et al. [14]
**Serum crosslaps**	2	Li et al. [13]<br>Solau-Gervais et al. [20]
**Osteocalcin**	2	Li et al. [13]<br>Solau-Gervais et al. [20]
**Other blood markers (endothelin 1, VEGF, EGF, calcium, phosphorus, creatinine, alkaline phosphatase, lipid profile, RF, ANA, TNF)**	3	Wu et al. [25]<br>Li et al. [13]<br>Przepiera-Będzak et al. [18]
**Undefined laboratory markers**	1	Maccora et al. [16]
**Imaging and other investigations**		
Radiographic evidence of osteoarticular manifestation (osteitis, hyperostosis, sacroiliitis)	3	Zwaenepoel et al. [28]<br>Amital et al. [2]<br>Skrabl-Baumgartner et al. [19]
**CT evidence of osteoarticular manifestation (osteitis, hyperostosis)**	2	Amital et al. [2]<br>Skrabl-Baumgartner et al. [19]
**Bone scintigraphy uptake**	1	Amital et al. [2]
**Bone biopsy**	2	Amital et al. [2]<br>Wu et al. [25]

**Other evidence of synovitis**	2	Amital et al. [2]<br>Wendling et al. [24]
**MRI evidence of osteoarticular manifestation (osteitis including bone marrow oedema, osteal erosions, or synovitis, +/- joint effusion; muscle oedema, subcutaneous soft tissue swelling, periosteitis, synovial thickening; sacroiliitis)**	6	Li et al. [13]<br>Assmann et al. [3]<br>Zwaenepoel et al. [28]<br>Wang et al. [23]<br>Jung et al. [9]
**CBCT, changes in area of cortical or medullary lysis**	1	Wang et al. [23]
**BMI**	1	Przepiera-Będzak et al. [18]
**Waist-hip ratio**	1	Przepiera-Będzak et al. [18]

HAS outcome measure is reported as presented on original manuscript [3]

Abbreviations: VAS - Visual Analogue Scale; NSAIDs - Non-steroidal Anti-inflammatory Drugs; NAPSI - Nail Psoriasis Severity Index; PPPASI - Palmoplantar Pustulosis Area and Severity Index; PASI – Psoriasis Area and Severity Index; BASFI - Bath Ankylosing Spondylitis Functional Index; HAQ - Health Assessment Questionnaire; BASMI - Bath Ankylosing Spondylitis Metrology Index; ASQoL - Ankylosing Spondylitis Quality of Life; EQ-5D - EuroQol-5D; SF-36 - 36-Item Short Form Survey; SF-8 – Short Form 8; DLQI - Dermatology Life Quality Index; BASDAI - Bath Ankylosing Spondylitis Disease Activity Index; ASDAS - Ankylosing Spondylitis Disease Activity Score; PGA - Physician Global Assessment; HAS - Health Assessment Score; ESR - Erythrocyte Sedimentation Rate; CRP - C-reactive Protein; hs-CRP - high sensitivity C-reactive Protein; VEGF - Vascular Endothelial Growth Factor; EGF - Endothelial Growth Factor; RF - Rheumatoid Factor; ANA - Antinuclear Antibody; TNF - Tumour Necrosis Factor; CT - Computed Tomography; CBCT - Cone Beam Computed Tomography; MRI - Magnetic Resonance Imaging; BMI - Body Mass Index.

Table S4: Study design summary

Study reference	Study design	Number of subjects	Inclusion criteria	Exclusion criteria	Study duration
Aljuhani et al **[1]**	retrospective single-centre study	41	Benhamou et al.	children	median follow up 30 months (range 5.5 - 102 months)
Amital et al **[2]**	open-label trial	10	• Benhamou et al.<br>• refractory to other medications	As per Benhamou et al.	mean follow up 24 ± 15 months (maximum 38 months)
Assmann et al **[3]**	prospective interventional study	30	• Chamot et al. and Kahn et al.<br>• >18 years old with clinical activity of SAPHO syndrome	• Patients with axial disease & spondylitis<br>• antibiotics treatment in previous 12 weeks<br>• pregnant or breastfeeding women<br>• patients on medications with corticosteroids in higher dose than 10mg prednisolone or equivalent steroids per day<br>• psychiatric disorders that may compromise therapy compliance<br>• contraindications for administration of azithromycin, doxycycline, + clindamycin (including	28 weeks

				history of allergy to any antibiotics)	
Abdelghani et al **[4]**	observational - case series	6	• SAPHO syndrome diagnosis by clinical & radiologic criteria (bone radiography, axial CT, and scintigraphy)<br>• painful clinical progression despite therapy with several NSAIDs, associated with at least 1 of: MTX, pamidronate, antibiotics.	Undisclosed	Undisclosed
**Colina et al [5]**	observational - case series (prospective)	14	• Benhamou et al.<br>• refractory to NSAIDs, glucocorticoids, and DMARDs	Undisclosed	mean follow up 5.4 years (range 7 months - 10.5 years)
**Guignard et al [6]**	retrospective chart review	5	• radiological and bone scan evidence of SAPHO syndrome, and histological exam of bone and/or skin lesion specimens confirming diagnosis<br>• pain refractory to NSAIDs	Undisclosed	mean follow up 2.8 years (range 6 months - 5 years)
**Hayem, G et al [7]**	retrospective single-centre study	120	Benhamou et al.	As per Benhamou et al.	mean follow up 4.9 years (range 1 - 23 years)
**Huang et al [8]**	retrospective observational	24	Modified Kahn et al. (2003)	As per Kahn et al. (2003)	mean follow up 2.5 years (range

					6 months - 7 years)
**Jung, J et al [9]**	clinical trial	10	• Chamot et al.<br>• Kahn et al.<br>• symptomatic osteitis in sternocostoclavicular region	Undisclosed	12 weeks ± 1wk
**Kerrison, C et al [10]**	retrospective observational study	7	• Kahn MF<br>• refractory to conventional therapy (analgesia, NSAIDs, corticosteroids, MTX)	Undisclosed	median (range) follow up 20 months (9-31 months)
**Li, C et al [11]**	case series: single-centre, open-label, single-arm, 12-week prospective pilot study	13	• Hayem et al<br>• nail lesions (baseline overall NAPSI >14) and active PPP (PPPASI ≥8 with at least 10% total surface of palms and soles affected)	Undisclosed	12 weeks
**Li, C et al [12]**	cross-sectional observational study	164 (41/164 receiving TNFi)	Kahn & Khan's criteria	Undisclosed	Undisclosed
**Li, C et al [13]**	prospective open clinical trial	30	• age 18-70<br>• diagnostic criteria reported by Nguyen in 2012<br>• spinal BMO on MRI<br>• normal routine serum test results<br>• consent to treatment and follow up for at least 1 year	pregnant or lactating women	Undisclosed

**Hurtado-Nedelec, M et al [14]**	open clinical trial	29 (3/29 receiving etanercept)	Benhamou et al criteria with at least one typical osseous involvement	Undisclosed	28 days of etanercept therapy
**Maatallah, K et al [15]**	retrospective 2-centre study	23	Benhamou et al criteria	Undisclosed	average (range) follow up 4 years (1-8 years)
**Maccora, I et al [16]**	case series	5	diagnosis at Rheumatology Unit from March 2015-June 2020	Undisclosed	Undisclosed
**Matzaroglou, Ch et al [17]**	case series	5	Benhamou et al.	As per Benhamou et al.	Undisclosed
**Przepiera-Będzak, H et al [18]**	case control	46<br>NB. 30 healthy controls	Kahn criteria	Undisclosed	Undisclosed
**Skrabl-Baumgartner, A et al [19]**	Retrospective single-centre comparative study	10	SAPHO syndrome diagnosis and treatment at Medical University of Graz from March 2004 - September 2016, with at least 6 months follow up	Undisclosed	median follow up 4.3 years
**Solau-Gervais, E et al [20]**	open-label	13	• Benhamou et al criteria<br>• VAS >40mm<br>• refractory to NSAIDs<br>• had stopped 2nd-line drugs and steroids for at least 3 months<br>• taking NSAIDs and/or analgesics at stable dose	Undisclosed	6 months follow up
**Van Doornum, S et al [21]**	retrospective review	6	Benhamou et al.	As per Benhamou et al.	Undisclosed
**Wang, L et al [22]**	single-centre clinical trial	30	• 18–70 years of age<br>• Kahn (2003) criteria<br>• ASDAS on basis of CRP level ≥ 1.3	• pregnant or lactating women<br>• with a desire to have children	12 weeks

			• VAS in global osteoarticular pain ≥ 4	• glucocorticoids, cDMARDs or biological agent treatment within 3 months before recruitment<br>• cardiac, hepatic, renal dysfunction<br>• significant haematology abnormalities (including anaemia, thrombocytopenia, leukopenia)<br>• definite history of chronic serious infection, or any recurrent infection or cancer.	
**Wang, M et al [23]**	retrospective	26	• Kahn (2003) criteria<br>• mandibular involvement	Undisclosed	median follow up 2.1 years (mean 2.1, range 0.1-6.0 years)
**Wendling, D et al [24]**	case series: monocentric retrospective observational analysis	5	• Benhamou et al criteria<br>• refractory to previous treatments	Undisclosed	mean treatment duration 5.5 months
**Wu, N et al [25]**	case series: single-center, retrospective study	24	• Modified Kahn criteria<br>• Onset age 18 years old or younger	Undisclosed	mean follow up period 39.2 ± 15.2 months
**Xiang, Y et al [26]**	retrospective	58	• Kahn criteria<br>• at least 18 years old	Undisclosed	Undisclosed

**Yap, F et al [27]**	Retrospective single-geographic regional cohort study	21	Kahn and Khan criteria	Undisclosed	median follow up 6 years (range 2-32 years)
**Zwaenepoel, T et al [28]**	retrospective case series	21	Kahn and Khan criteria	Undisclosed	median follow up duration 45 months (range 0-188 months)

Abbreviations: CT - Computed Tomography; NSAIDs - Non-steroidal Anti-inflammatory Drugs; MTX – methotrexate; DMARD – Disease Modifying Antirheumatic Drug; PPP – palmoplantar pustulosis; NAPSI - Nail Psoriasis Severity Index; PPPASI - Palmoplantar Pustulosis Area and Severity Index; BMO – bone marrow oedema; VAS – Visual Analogue Score; ASDAS - Ankylosing Spondylitis Disease Activity Score; CRP - C-reactive Protein.

Table S5: Treatments investigated.

Drug Classification	Drugs	Outcomes	Adverse effects	Total recipients	References
NSAIDs	celecoxib<br>loxoprofen<br>diclofenac<br>etoricoxib<br>naproxen<br>ibuprofen<br>piroxicam<br>lornoxicam	• variable responses: inadequate to complete improvement, with minority achieving partial or full remission [1, 7, 15, 16, 19, 25, 28]<br>• limited efficacy as first line [21]<br>• some report efficacy in reducing disease efficacy [18]<br>• positive symptomatic alleviation seen as part of combination treatment [8, 17]	• Gastrointestinal upset [7, 21]<br>• Loss of efficacy [21]	295	• Wu et al. [25]<br>• Matzaroglou et al. [17]<br>• Maccora et al. [16]<br>• Huang et al. [8]<br>• Zwaenepoel et al. [28]<br>• Hayem et al. [7]<br>• Aljuhani et al. [1]<br>• Maatallah et al. [15]<br>• Skrabl-Baumgartner et al. [19]<br>• Van Doornum et al. [21]<br>• Przepiera-Będzak et al. [18]
Corticosteroids	Prednisone<br>hydrocortisone<br>prednisolone<br>triamcinolone	• injections effective in several studies [1, 7, 9]<br>• partial improvement with oral therapy [1, 7]<br>• hydrocortisone showed partial response in one patient [25]<br>• transient efficacy with systemic steroids [19]<br>• corticosteroids show benefit with other	20mg triamcinolone injections well-tolerated [9]	56	• Wu et al. [25]<br>• Maccora et al. [16]<br>• Hayem et al. [7]<br>• Wang et al. [23]<br>• Aljuhani et al. [1]<br>• Skrabl-Baumgartner et al. [19]<br>• Jung et al. [9]

		therapy, including of cutaneous manifestations with topical application [16, 23]			
Conventional DMARDs	methotrexate<br>sulfasalazine<br>leflunomide<br>azathioprine<br>cyclosporin<br>Cyclophosphamide<br>apremilast<br>hydroxychloroquine<br>Colchicine	• generally variable responses [28]<br>• Colchicine: little to no improvement [1, 7]<br>• Sulfasalazine: mild to partial improvement, including osteoarticular benefit in some refractory to NSAIDs, and some having sustained long-term benefit [1, 7, 15, 27]<br>• Methotrexate: effective in combination therapy; effective in some for osteoarticular and cutaneous manifestations; some received methotrexate but switched to other cDMARDs without benefit, while some received methotrexate monotherapy and benefitted [1, 7, 15, 19, 27]	• methotrexate and sulfasalazine generally well-tolerated [7]<br>• methotrexate may be associated with metabolic syndrome, with significantly higher BMI and lipid profiles than those not treated with methotrexate [18]	173	• Maccora et al. [16]<br>• Huang et al. [8]<br>• Zwaenepoel et al. [28]<br>• Hayem et al. [7]<br>• Wang et al. [23]<br>• Aljuhani et al. [1]<br>• Maatallah et al. [15]<br>• Skrabl-Baumgartner et al. [19]<br>• Yap et al. [27]<br>• Przepiera-Będzak et al. [18]

		• Combination treatment had mixed efficacy, with DMARDs showing partial or good effects in majority alongside NSAIDs, but some not benefitting despite receiving a number of therapies including at least one cDMARD [7, 8, 23]			
TNF inhibitors	Infliximab<br>Adalimumab<br>Etanercept<br>certolizumab	• mostly positive (partial or complete) clinical responses, including reduced pain and disease activity, reduced osteoarticular presentation, improved dermatological manifestation [1, 4, 8, 12, 14-16, 25, 27, 28]<br>• inefficacy was less frequently reported [19]<br>• may be useful as part of combination therapy: in some where NSAIDs and methotrexate were ineffective, add on anti TNF was effective [8]	several cases of new or exacerbated skin lesions (paradoxical psoriasis, urticaria, psoriasiform lesions, worsened palmoplantar pustulosis) reported [4, 12]	90	• Wu et al. [25]<br>• Maccora et al. [16]<br>• Huang et al. [8]<br>• Zwaenepoel et al. [28]<br>• Aljuhani et al. [1]<br>• Maatallah et al. [15]<br>• Skrabl-Baumgartner et al. [19]<br>• Yap et al. [27]<br>• Hurtado-Nedelec et al. [14]<br>• Ben Abdelghani et al. [4]<br>• Li et al. [12]

Interleukin-targeted treatment	ustekinumab (anti-IL-12/23)<br>secukinumab (anti-IL-17A)<br>ixekizumab (anti-IL17A)	variable but generally negative outcomes with interleukin-targeted treatment: IL6 inhibitor alone insufficient to remit bone lysis, poor outcomes with secukinumab and ixekizumab, lack of major rheumatic symptomatic alleviation despite some experiencing skin improvement with ustekinumab or secukinumab [23, 24, 27]	2 cases of paradoxical psoriasis flares [24]	9	• Wang et al. [23]<br>• Yap et al. [27]<br>• Wendling et al. [24]
JAK inhibitors	tofacitinib	dermatological manifestation and associated quality of life improvement, with transient pain reduction [11]	no severe adverse events [11]	13	Li et al. [11]

Bisphosphonates	Pamidronate<br>Alendronate<br>Zoledronic acid	• generally useful for osteoarticular manifestations, including in refractory cases, with pain reduction, reduced need for other medication, and functional improvement [1, 2, 5-8, 10, 13, 15, 19-21, 23, 25, 28]<br>• few reports of unsustained benefit [27]<br>• no convincing benefit for cutaneous manifestations overall [2, 5]	• generally well-tolerated, no severe adverse effects [5, 13]<br>• fever or flu-like presentation associated with initial infusion(s) [1, 6, 10, 13, 16]<br>• less reported adverse effects include gastrointestinal discomfort, transient headache, hypocalcaemia [8, 10, 13]	176	• Wu et al. [25]<br>• Kerrison et al. [10]<br>• Maccora et al. [16]<br>• Huang et al. [8]<br>• Li et al. [13]<br>• Zwaenepoel et al. [28]<br>• Hayem et al. [7]<br>• Wang et al. [23]<br>• Aljuhani et al. [1]<br>• Amital et al. [2]<br>• Colina et al. [5]<br>• Guignard et al. [6]<br>• Maatallah et al. [15]<br>• Skrabl-Baumgartner et al. [19]<br>• Solau-Gervais et al. [20]<br>• Van Doornum et al. [21]<br>• Yap et al. [27]
Antimicrobials	Azithromycin<br>Doxycycline<br>Clindamycin<br>Tetracycline	• predominantly ineffective with negative bacteriological studies [1, 7, 15]<br>• some experience improved osteitis and skin lesions including alongside NSAID treatment [3, 17]	3 reports of non-descript intolerance [1, 3]	85	• Matzaroglou et al. [17]<br>• Assmann et al. [3]<br>• Hayem et al. [7]<br>• Aljuhani et al. [1]<br>• Maatallah et al. [15]<br>• Przepiera-Będzak et al. [18]

Other	Tripterygium wilfordii	varied results including with combination therapy: ineffective in some, effective in others [8, 22, 23]	one case of severe alanine aminotransferase elevation; most common non-serious adverse effects were controllable ALT elevation, no irregular menstruation [22]	41	• Huang et al. [8]<br>• Wang et al. [23]<br>• Wang et al. [22]

Table S6: Evidence level.

Reference	Date published	Study type	Paper strengths	Paper limitations	Evidence level
**Aljuhani et al [1]**	02/01/2015	retrospective single-centre study	• relatively long study duration (median follow up 30 months, maximum 102 months)<br>• results consistent overall with existing series	• retrospective design<br>• subjective outcome measures lacking detail around how efficacy is assessed<br>• descriptive pain scoring lacking any recognised psychometric measures such as VAS<br>• predominantly female (73.17%) and White cohort (97.6%) from one centre<br>• wide range of follow up durations (minimum 5.5 months)<br>• small cohort (n=41)	6
**Amital et al [2]**	24/02/2004	open-label	• prohibited drug use beyond pamidronate and NSAIDs	• small, predominantly female (70%) cohort (n=10), all of Jewish descent (Sephardic + Ashkenazi)<br>• open-label study design<br>• NSAID use permitted and not recorded<br>• subjective patient reported pain outcome<br>• relatively short follow up period (mean 24 months, maximum 38 months)	3

**Assmann et al [3]**	21/09/2009	prospective interventional study	• gender distribution balanced (Female 53.33%)<br>• outcomes assessed at fixed periods, via both subjective measures and objective values<br>• MRI reported by radiologist blinded to treatment<br>• treatment changes and concomitant treatment during antibiotic therapy prohibited<br>• results consistent with existing case reports and uncontrolled observations with antibiotic regimes in SAPHO patients	• 60% patients lost by end of study (loss to follow up, n=7; antibiotic intolerance, n=2; medication change, n=9)<br>• large number of patients treated without biopsy (53.33%)<br>• subjective outcome measures (physician assessment of skin disease and osteitis activity; patient reported HAS; MRI of osteitis lesion)<br>• study duration relatively short<br>• no placebo control group<br>• heterogeneity of disease duration (range 1-27 years)	3
**Abdelghani et al [4]**	08/01/2010	case series	• therapeutic response based on recognised measures, including BASDAI + BASFI	• small cohort (n=6) of solely females<br>• BASDAI + BASFI mightn't be appropriate for SAPHO<br>• Subjective outcome measure (VAS)<br>• maximal follow up 42 months is relatively short	6
**Colina et al [5]**	2009	case series; prospective	• therapeutic response based on recognised measures (VAS)<br>• prospective design	• small cohort (n=14) of predominantly females (71.43%)<br>• wide range of follow up durations (range 7 months - 10.5 years, mean 5.4 years)<br>• no statistical analysis (due to sample size)	6

				• Subjective outcome measure (VAS)	
**Guignard et al [6]**	07/09/2002	retrospective chart-review	• therapeutic response predominantly based on recognised measures (VAS)<br>• secondary efficacy criteria (analgesia or anti-inflammatory drug use; interval duration between exacerbations)	• small cohort (n=5) of predominantly females (80%)<br>• wide range of follow up durations (mean 2.8 years, range 6 months - 5 years)<br>• heterogeneous disease duration at treatment (mean 10.4 years, range 4-22 years)<br>• retrospective design<br>• Subjective outcome measure (VAS)	6
**Hayem et al [7]**	01/12/1999	retrospective single-centre study	• creation of a simple, reproducible efficacy index for evaluation<br>• relatively large sample size (n = 120)<br>• relatively balanced gender distribution (females 58.33%)<br>• more diverse ethnic makeup than existing studies (88.33% European White, 10% North African, 0.83% Black African, 0.83% French West Indies)<br>• little loss to follow up in prospective cohort (2.94%)<br>• statistical analysis enables statistical significance assessment	• heterogenous treatment (drug class and dosage)<br>• multiple simultaneous drug therapy<br>• retrospective data collection; much quantitative information regarding therapies missing<br>• wide range of follow up durations (mean 4.9 years, range 1 year - 23 years)<br>• predominantly White European cohort	6

**Huang et al [8]**	13/08/2020	observational, retrospective		• small cohort (n=24) of predominantly female patients (62.5%)<br>• wide range of follow up durations (mean 2.5 years, range 6 months - 7 years)<br>• retrospective design<br>• subjective outcome measures not fully quantifiable (degree of pain +/or cutaneous manifestation relief)	6
**Jung et al [9]**	09/05/2012	clinical trial	• patient demographics relatively balanced (F:M = 6:4)<br>• standard procedure of injection under ultrasound-guidance by same physician<br>• statistical analysis	• small cohort (n=10)<br>• short study duration 12 ± 1 week<br>• MRI imaging using T1 and T2, without fat-saturated techniques<br>• treatment heterogeneity following concomitant use of preexisting therapy	3
**Kerrison et al [10]**	07/06/2004	retrospective observational study	• standardised data collection method	• small solely female cohort (n=7)<br>• study duration short (median 20 months, range 9 - 31 months)<br>• retrospective study design<br>• descriptive, non-specific outcome measures<br>• heterogenous definitions and diagnostic criteria for SAPHO syndrome	6

				• heterogenous pamidronate therapeutic regimes in literature	
**Li et al [11]**	30/09/2020	case series: single-centre, open-label, single-arm, 12-week prospective pilot study	• statistical analysis<br>• primary therapeutic response based on recognised measures (change from baseline NAPSI)<br>• secondary efficacy criteria (change from baseline PPPASI scores, VAS for global osteoarticular pain, BASDAI scores, DLQI scores, inflammatory markers ESR and high-sensitivity CRP)	• open-label design<br>• small solely female (n=13) cohort from single centre<br>• study duration short (12 weeks)<br>• Only Asian individuals included in population<br>• Subjective outcome measure (VAS)	3
**Li et al [12]**	03/04/2018	cross-sectional observational study	• relatively larger study population given nature of disease<br>• statistical analyses performed	• retrospective design<br>• small cohort treated with anti-TNF agents (41/164)<br>• determined tissue & serum TNF-α levels only in minority (28.57%)<br>• no gender distribution data available<br>• solely Chinese population<br>• distinguishing paradoxical skin lesions from new lesions truly from SAPHO syndrome disease progression	6

**Li et al [13]**	2018	prospective clinical trial open study	• prospective study design<br>• inclusion criteria necessitated stable VAS score<br>• prohibited concomitant treatment for minimum 3 months, except recorded use of NSAIDs as necessary<br>• statistical analyses performed<br>• sample size estimation performed<br>• measures taken to account for missing post-baseline data (last-observation-carried-forward methodology)<br>• 2 experienced radiologists reviewed bone marrow oedema imaging against standardised methods<br>• therapeutic response based on recognised measures (including BASDAI, BASFI, ASDAS) and objective lab results (including ESR, hypersensitive CRP, osteocalcin, blood calcium, alkaline phosphatase, and β-crosslaps - more objective and may be more sensitive than VAS)	• small cohort (n=30) of predominantly females (66.67%)<br>• short follow up duration (1 year)<br>• open study lacking control group (due to lack of validated treatment guidelines and rarity of condition)<br>• Subjective outcome measure (VAS)	3
**Hurtado-Nedelec et al [14]**	19/06/2008	interventional; clinical trial	• use of similar groups to compare results against (rheumatoid arthritis patients, n=22; psoriatic arthritis patients, n=21; healthy controls, n=15)	• small cohort (n=29) of predominantly females (82.76%), of whom very few were eligible to receive etanercept therapy (n=3)	3

			• objective quantifiable outcome measures (total immunoglobulin and autoantibody levels in serum)<br>• statistical analyses performed	• short study duration (28 days of etanercept therapy)<br>• open study<br>• solely laboratory outcome measures	
**Maatallah et al [15]**	25/08/2021	retrospective 2-centre study	• use of CT alongside MRI<br>• no missing data in medical files<br>• few patients lost to follow-up<br>• fairly balanced sex distribution (females 60.87%) with diverse age range included (mean age ± standard deviation 44.7 ± 16.9 years, range 14–76 years)	• retrospective design<br>• small cohort (n=23)<br>• study duration short - average 4 years (range 1 - 8 years)<br>• concomitant treatment use	6
**Maccora et al [16]**	02/11/2021	case series		• small cohort (n=5), all of whom were Caucasian<br>• treatment heterogeneity of treatment (all received anti TNF agents, but some also received further treatment with IV bisphosphonate, or with topical corticosteroid)	6
**Matzaroglou et al [17]**	05/11/2009	case series		• small cohort (n=5)<br>• non-standardised descriptive outcome measures (resolution of initial presenting complaint)<br>• few had bone biopsy (done in 2/5 cases)	6
**Przepiera-Będzak et al [18]**	05/07/2018	case control	• healthy control volunteers (n=30)<br>• statistical analyses performed<br>• quantifiable measures to assess association with metabolic	• small cohort (n=46); predominantly female (86.96%) and all Caucasian<br>• Subjective outcome measure (VAS)	4

			syndrome (total cholesterol, HDL, LDL, triglycerides; BMI; waist-hip ratio) and NSAID efficacy (BASDAI, VAS, ESR, CRP, serum IL6, IL18, IL23, endothelin 1, VEGF, EGF)		
**Skrabl-Baumgartner et al [19]**	23/07/2019	Retrospective single-centre comparative study	• relatively balanced gender distribution (F:M = 6:4)<br>• statistical analyses performed<br>• bone specimens reevaluated by histopathologist to compare histopathologic results	• small cohort (n=10) across single-centre<br>• retrospective study design<br>• non-quantifiable outcome measures (remission/relapsing course/persistent course as defined by authors)	6
**Solau-Gervais et al [20]**	25/10/2005	open-label	• statistical analyses performed<br>• quantifiable outcome measures to evaluate treatment (ESR, CRP, serum crosslaps, osteocalcin)	• small cohort (n=13), predominantly female (92.31%)<br>• short follow up duration (6 months)<br>• wide range of disease duration at time of treatment (range 1-10 years)<br>• Subjective outcome measure (VAS)	3
**Van Doornum et al [21]**	25/05/2002	retrospective review		• small (n=6) solely female cohort<br>• retrospective study design including patient identification by physician recall	6

**Wang et al [22]**	03/01/2021	single-centre clinical trial	• relatively balanced gender distribution (F:M = 18:12)<br>• use of widely used patient-reported outcomes & laboratory tests for axial spondyloarthritis (including ASDAS, BASDAI, HAQ-S, ASQoL, ESR + hsCRP, VAS)<br>• randomised allocation to dose groups using a computer-generated random allocation sequence<br>• statistical analyses performed<br>• prohibited concomitant treatment (glucocorticoids, cDMARDs, biological agents), except monitored use of NSAIDs as necessary	• small cohort (n=30) across a single centre<br>• short follow up duration (12 weeks)<br>• lack of placebo control<br>• lack of imaging to assess therapeutic efficacy<br>• NSAID use not prohibited<br>• Subjective outcome measure (VAS)	3
**Wang et al [23]**	11/02/2020	Retrospective	• use of recognised and commonly used imaging modalities (CBCT and MRI, each requiring little or no radiation respectively) - objective outcome measures<br>• statistical analyses performed<br>• diagnosis as per Kahn MF criteria assessed by multiple professionals	• small cohort (n=26), predominantly female (61.54%)<br>• most patients not treatment-naïve<br>• one individual received multiple concomitant drug treatment<br>• short follow-up duration (mean 2.1 years, range 0.1-6.0 years)<br>• retrospective study design; not all patients had baseline & follow-up MRI & CBCT data	6

**Wendling et al [24]**	24/05/2017	case series: monocentric retrospective observational analysis	• mixture of subjective and more objective comprising outcome measures (PGA, BASDAI, number of synovitis, PPPASI-50, global tolerance)	• small cohort (n=5), predominantly female (80%)<br>• retrospective study design<br>• short follow up duration (mean treatment duration 5.5 months)<br>• no specific inclusion criteria<br>• lack of SAPHO-specific evaluation tools<br>• heterogenous treatment doses	6
**Wu et al [25]**	15/09/2020	case series: single-center, retrospective study	• statistical analyses performed	• small predominantly male (62.5%) Chinese cohort (n=24) from a single centre<br>• retrospective study design and heterogenous data collection methodology<br>• short follow up duration (mean follow up 39.2 ± 15.2 months)<br>• cases seen may be especially clinically severe considering centre conducted at being a renowned national Chinese medical centre<br>• heterogeneity in defining SAPHO syndrome by diagnostic criteria<br>• non-quantifiable outcome measures (degree of response based on complete/partial elimination of clinical	6

				manifestations + lab or imaging abnormality, or worsening)	
**Xiang et al [26]**	27/01/2021	Retrospective	• outcome measures widely used (VAS, PPPASI, NAPSI)<br>• statistical analyses performed	• precursory exploration of tonsillitis prevalence & characteristics<br>• small cohort (n=58) from one centre<br>• included patients with tonsillitis history related to SAPHO (may introduce recall bias)<br>• retrospective study design<br>• lack of control group<br>• Subjective outcome measure (VAS)	6
**Yap et al [27]**	05/10/2021	Retrospective single-geographic regional cohort study	• long follow up duration (median 6 years, maximum 32 years)<br>• statistical analyses<br>• results consistent with those reported in China + Europe	• retrospective study design with use of consultant recall and inconsistent data collection including potential confounders<br>• imaging wasn't routine - can't report exact details of skeletal damage with or without treatment over time<br>• small predominantly Caucasian (95%) cohort (n=21) from a single geographical area<br>• heterogeneous therapy	6

				• no long-term structured evaluation of skeletal / joint tissues<br>• subjective outcome measures	
**Zwaenepoel et al [28]**	25/04/2016	retrospective case series		• retrospective study<br>• small cohort (n=21) of predominantly females (76.19%)<br>• non-quantifiable, subjective outcome measures (full or partial remission of osteoarticular manifestations as judged by expert opinion)<br>• wide range of follow up durations (range 0 - 188 months, median 45 months)	6

References

1. Aljuhani F, Tournadre A, Tatar Z, Couderc M, Mathieu S, Malochet-Guinamand S, et al. The SAPHO syndrome: a single-center study of 41 adult patients. J Rheumatol. 2015;42(2):329-34.
2. Amital H, Applbaum YH, Aamar S, Daniel N, Rubinow A. SAPHO syndrome treated with pamidronate: an open-label study of 10 patients. Rheumatology (Oxford). 2004;43(5):658-61.
3. Assmann G, Kueck O, Kirchhoff T, Rosenthal H, Voswinkel J, Pfreundschuh M, et al. Efficacy of antibiotic therapy for SAPHO syndrome is lost after its discontinuation: an interventional study. Arthritis Res Ther. 2009;11(5):R140.
4. Ben Abdelghani K, Dran DG, Gottenberg JE, Morel J, Sibilia J, Combe B. Tumor necrosis factor-alpha blockers in SAPHO syndrome. J Rheumatol. 2010;37(8):1699-704.
5. Colina M, La Corte R, Trotta F. Sustained remission of SAPHO syndrome with pamidronate: a follow-up of fourteen cases and a review of the literature. Clin Exp Rheumatol. 2009;27(1):112-5.
6. Guignard S, Job-Deslandre C, Sayag-Boukris V, Kahan A. Pamidronate treatment in SAPHO syndrome. Joint Bone Spine. 2002;69(4):392-6.
7. Hayem G, Bouchaud-Chabot A, Benali K, Roux S, Palazzo E, Silbermann-Hoffman O, et al. SAPHO syndrome: a long-term follow-up study of 120 cases. Semin Arthritis Rheum. 1999;29(3):159-71.
8. Huang H, Zhang Z, Zhao J, Hao Y, Zhou W. The effectiveness of treatments for patients with SAPHO syndrome: a follow-up study of 24 cases from a single center and review of literature. Clin Rheumatol. 2021;40(3):1131-9.
9. Jung J, Molinger M, Kohn D, Schreiber M, Pfreundschuh M, Assmann G. Intra-articular glucocorticosteroid injection into sternocostoclavicular joints in patients with SAPHO syndrome. Semin Arthritis Rheum. 2012;42(3):266-70.
10. Kerrison C, Davidson JE, Cleary AG, Beresford MW. Pamidronate in the treatment of childhood SAPHO syndrome. Rheumatology (Oxford). 2004;43(10):1246-51.
11. Li C, Li Z, Cao Y, Li L, Li F, Li Y, et al. Tofacitinib for the Treatment of Nail Lesions and Palmoplantar Pustulosis in Synovitis, Acne, Pustulosis, Hyperostosis, and Osteitis Syndrome. JAMA Dermatol. 2021;157(1):74-8.
12. Li C, Wu X, Cao Y, Zeng Y, Zhang W, Zhang S, et al. Paradoxical skin lesions induced by anti-TNF-α agents in SAPHO syndrome. Clin Rheumatol. 2019;38(1):53-61.
13. Li C, Zhao Y, Zuo Y, Zhou Y, Zhang F, Liu S, et al. Efficacy of bisphosphonates in patients with synovitis, acne, pustulosis, hyperostosis, and osteitis syndrome: a prospective open study. Clin Exp Rheumatol. 2019;37(4):663-9.
14. Hurtado-Nedelec M, Chollet-Martin S, Nicaise-Roland P, Grootenboer-Mignot S, Ruimy R, Meyer O, et al. Characterization of the immune response in the synovitis, acne, pustulosis, hyperostosis, osteitis (SAPHO) syndrome. Rheumatology (Oxford). 2008;47(8):1160-7.
15. Maatallah K, Zouaoui K, Ferjani H, Dhahri R, Metoui L, Ben Nessib D, et al. The Synovitis, Acne, Pustulosis, Hyperostosis, And Osteitis (SAPHO) syndrome: A two-center study of 23 patients. Egyptian Rheumatologist. 2022;44(1):41-6.
16. Maccora I, Marrani E, Maniscalco V, Mastrolia MV, Pagnini I, Simonini G. Diagnostic challenge of synovitis, acne, pustulosis, hyperostosis, and osteitis (SAPHO) syndrome in pediatric age: A monocentric case series. Mod Rheumatol. 2021;31(6):1228-31.

17. Matzaroglou C, Velissaris D, Karageorgos A, Marangos M, Panagiotopoulos E, Karanikolas M. SAPHO Syndrome Diagnosis and Treatment: Report of Five Cases and Review of the Literature. Open Orthop J. 2009;3:100-6.
18. Przepiera-Będzak H, Fischer K, Brzosko M. Serum interleukin-23 protects, whereas methotrexate treatment stimulates selected components of the metabolic syndrome in patients with SAPHO syndrome. Arch Med Sci. 2021;17(1):120-6.
19. Skrabl-Baumgartner A, Singer P, Greimel T, Gorkiewicz G, Hermann J. Chronic non-bacterial osteomyelitis: a comparative study between children and adults. Pediatr Rheumatol Online J. 2019;17(1):49.
20. Solau-Gervais E, Soubrier M, Gerot I, Grange L, Puechal X, Sordet C, et al. The usefulness of bone remodelling markers in predicting the efficacy of pamidronate treatment in SAPHO syndrome. Rheumatology (Oxford). 2006;45(3):339-42.
21. Van Doornum S, Barraclough D, McColl G, Wicks I. SAPHO: rare or just not recognized? Semin Arthritis Rheum. 2000;30(1):70-7.
22. Wang L, Gong L, Zhang X, Cao Y, Long P, Zhang W, et al. Tripterygium wilfordii Hook F. in the treatment of synovitis, acne, pustulosis, hyperostosis, and osteitis syndrome: a clinical trial. Clin Rheumatol. 2021;40(6):2427-38.
23. Wang M, Li Y, Cao Y, Lu X, Liu Y, Zhao J, et al. Mandibular involvement in SAPHO syndrome: a retrospective study. Orphanet J Rare Dis. 2020;15(1):312.
24. Wendling D, Aubin F, Verhoeven F, Prati C. IL-23/Th17 targeted therapies in SAPHO syndrome. A case series. Joint Bone Spine. 2017;84(6):733-5.
25. Wu N, Shao Y, Huo J, Zhang Y, Cao Y, Jing H, et al. Clinical characteristics of pediatric synovitis, acne, pustulosis, hyperostosis, and osteitis (SAPHO) syndrome: the first Chinese case series from a single center. Clin Rheumatol. 2021;40(4):1487-95.
26. Xiang Y, Wang Y, Cao Y, Li Z, Xiong D, Wang L, et al. Tonsillitis as a possible predisposition to synovitis, acne, pustulosis, hyperostosis and osteitis (SAPHO) syndrome. Int J Rheum Dis. 2021;24(4):519-25.
27. Yap FHX, Olsson-White D, Roddy J, Cook NJ, Langlands DR, Manners PJ, et al. Long-term Clinical Outcomes in Synovitis, Acne, Pustulosis, Hyperostosis, and Osteitis Syndrome. Mayo Clin Proc Innov Qual Outcomes. 2021;5(3):574-82.
28. Zwaenepoel T, Vlam K. SAPHO: Treatment options including bisphosphonates. Semin Arthritis Rheum. 2016;46(2):168-73.
